# Combination therapy versus monotherapy: a randomised pilot study on the evolution of inflammatory parameters after ventilator associated pneumonia [ISRCTN31976779]

**DOI:** 10.1186/cc4879

**Published:** 2006-03-28

**Authors:** Pierre Damas, Christophe Garweg, Mehran Monchi, Monique Nys, Jean-Luc Canivet, Didier Ledoux, Jean-Charles Preiser

**Affiliations:** 1 Department of General Intensive Care, University Hospital Centre, Domaine universitaire du Sart-Tilman, B-4000 Liege, Belgium

## Abstract

**Introduction:**

Combination antibiotic therapy for ventilator associated pneumonia (VAP) is often used to broaden the spectrum of activity of empirical treatment. The relevance of such synergy is commonly supposed but poorly supported. The aim of the present study was to compare the clinical outcome and the course of biological variables in patients treated for a VAP, using a monotherapy with a beta-lactam versus a combination therapy.

**Methods:**

Patients with VAP were prospectively randomised to receive either cefepime alone or cefepime in association with amikacin or levofloxacin. Clinical and inflammatory parameters were measured on the day of inclusion and thereafter.

**Results:**

Seventy-four mechanically ventilated patients meeting clinical criteria for VAP were enrolled in the study. VAP was microbiologically confirmed in 59 patients (84%). Patients were randomised to receive cefepime (C group, 20 patients), cefepime with amikacin (C-A group, 19 patients) or cefepime with levofloxacin (C-L group, 20 patients). No significant difference was observed regarding the time course of temperature, leukocytosis or C-reactive protein level. There were no differences between length of stay in the intensive care unit after infection, nor in ventilator free days within 28 days after infection. No difference in mortality was observed.

**Conclusion:**

Antibiotic combination using a fourth generation cephalosporin with either an aminoside or a fluoroquinolone is not associated with a clinical or biological benefit when compared to cephalosporin monotherapy against common susceptible pathogens causing VAP.

## Introduction

Ventilator associated pneumonia (VAP) is the most frequent nosocomial infection in the intensive care unit (ICU) [1,2], is a major determinant for increases in ICU length of stay and ventilator days [3] and is associated with a two-fold increase in the mortality rate [4]. In contrast to the importance of starting therapy promptly, as any delay increases mortality, morbidity and cost [1,5,6], the initial choice of antibiotic is still a matter of debate. In particular, the use of a combination therapy versus a monotherapy, especially against particular strains such as *Pseudomonas aeruginosa*, remains controversial. In a recent meta-analysis, no benefit was found for combination therapy over monotherapy in terms of mortality or prevention of resistant germs [7].

Combination therapy is actually proposed not only to broaden the antibacterial spectrum to each potential pathogen, but also to raise the bactericidal activity of the treatment. If it is difficult to demonstrate an effect in terms of mortality [7], other outcome variables, including the evolution of inflammatory parameters and ventilator dependence, could be used to assess the effectiveness of different regimens. Temperature, leukocytosis and PaO_2_/FiO_2 _(arterial oxygen tension/inspiratory oxygen fraction) are in fact part of the clinical pulmonary infection score (CPIS) used to define the presence of VAP and to follow the response to treatment [8,9]. In addition to these parameters, C-reactive protein (CRP), a sensitive marker of inflammation produced by the liver in response to cytokines released by activated mononuclear phagocytes cells [10], is also used for the diagnosis and monitoring of different acute inflammatory and infectious processes [11,12]. In community acquired pneumonia (CAP), it was confirmed to be a useful marker for the differentiation of bacterial and viral infections [13-15]. It also allows an evaluation of the adequacy of antibacterial treatment as the CRP plasma levels decreased by 50% within 3.3 days in patients receiving adequate treatment [16]. One can wonder whether this advantage holds true for VAP.

The aim of this study, therefore, was to evaluate the clinical evolution of patients with VAP who were treated empirically with one or two drugs, comparing as the main outcome the time course of clinical inflammatory parameters and duration of ventilatory support, and mortality as a secondary outcome. The *a priori *hypothesis is that synergistic action of combination therapy will accelerate the resolution of inflammation.

## Materials and methods

This prospective and randomised study was performed in the 26-bed general ICU of the University Hospital of Liege Sart-Tilman, Belgium, over a total period of 21 months, from April 2002 to December 2003. The study received the approval of the Ethics Committee of the hospital. Patients or their next of kin had to give their written informed consent.

### Patients

Patients were eligible for this study if they were older than 18 years, were mechanically ventilated for more than 48 hours and developed clinical evidence of VAP as defined by new and persistent radiographic infiltrate for at least 48 hours with at least three of the following: body temperature >38°C or <36°C; white blood cells >10,000 mm^3 ^or <4,000 mm^3^; macroscopically purulent tracheal aspirate; increase in CRP level of at least 50 mg/l within the last 24 hours. The PaO_2_/FiO_2 _ratio was also obtained in order to calculate a modified version of the CPIS [15], with a score superior to 6 considered as a high probability of VAP (Table 1). In addition, VAP had to be confirmed by culture of pathogens from the tracheal aspirate. Quantitative bacteriology was not required but, when obtained, growth of ≥ 10^5 ^in tracheal aspirate or 10^4 ^in bronchoalveolar lavage confirmed VAP. Samples for bacteriological cultures were also obtained between the third and fifth day of treatment and at the end of the eight to ten days' treatment.

Exclusion criteria included: patients already treated for another infection or having received antibiotic treatment during the last 15 days; patients with organ transplantation or suffering from hematological malignancy; and patients with a life expectancy of less than two days.

Eligible patients were further characterized by age, sex, underlying diseases, length of stay, length of ventilatory support before and after VAP, APACHE II score at the entry into the ICU and sequential organ failure assessment (SOFA) score at the beginning and during the course of VAP.

### Antibiotic treatment

Patients were randomised into three groups. The first (group C) received cefepime only (2 g every 8 hours) for 8 to 10 days; this dose was reduced if necessary according to the clearance of creatinine. The second (group C-A) received cefepime combined with amikacin (20 mg/kg, once daily) for 5 days, with adaptation to the level of the clearance of creatinine by increasing the delay between doses. The third (group C-L) received cefepime associated with levofloxacin (750 mg once daily) for 8 to 10 days. Cefepime could be changed to a narrower spectrum beta-lactamine in the case of susceptible agent or to imipenem in the case of resistance; amikacin or levofloxacin were kept during the entire course except if the pathogen was found to be resistant. The attending physician could overrule the protocol in the case of multidrug resistance.

CRP was measured daily in serum samples by an automated latex-enhanced turbidimetric assay with an analyzer Modular (Roche Hitachi Vilvoord, Belgium) for at least eight days.

### Outcome criteria

The efficacy of treatments was evaluated during treatment by the evolution in inflammatory parameters: PaO_2_/FiO_2_, temperature, leukocytosis and CRP level were measured each day for eight days. The improvement or worsening of the patient was also assessed by the change in SOFA score [16]. Eradication from or persistence of bacteria in tracheal aspirate was documented. Mortality at 28 days after diagnosis was used. The 28 ventilator free days (VFPs) after the diagnosis of VAP and the ICU length of stay were further obtained in order to compare the treatments used in the three groups.

### Statistical analyses

The time course of temperature, PaO_2_/FiO_2_, CRP and leukocytosis were compared by ANOVA for repeated values. Mechanical ventilation durations were compared by the Kaplan-Meier method. Baseline characteristics of patients were compared with the unpaired *t *test or the Wilcoxon rank sum test for continuous variables, depending on their distributions. Differences were considered significant if the *p* value was below 0.05. This study was a first attempt to evaluate the magnitude of the eventual differences between treatments in order to calculate a sufficient sample size to further demonstrate statistically significant differences if needed.

## Results

Seventy-four patients fulfilling the clinical VAP criteria were randomised into three groups. Of these, 24 patients received cefepime only (group C), 26 received cefepime with amikacin (group C-A) and 24 received cefepime with levofloxacin (group C-L).

Pneumonia was not microbiologically confirmed in 15 of these patients: 4 in the C group, 7 in the C-A group and 4 in the C-L group. Quantitative cultures were obtained in 34 patients (12, 12 and 10 for the C, C-A and C-L groups, respectively). The mean CPIS of these 34 patients (10.1 ± 1.7) was not significantly different from those without quantitative culture (9.5 ± 1.9). The mean CPIS of the 15 patients in whom VAP was not confirmed was significantly lower (6.3 ± 2.1).

The characteristics of the 59 patients in whom VAP was confirmed are given in Table 2. There were no significant differences between the three groups in terms of age, sex, underlying diseases, APACHE II and SOFA scores, CPIS, length of stay in the hospital before infection and duration of mechanical ventilation before VAP.

Eighty two bacteria strains were isolated from tracheal aspirate or bronchoalveolar lavage samples; 2 bacteria strains were isolated from 23 patients and only 1 from the remaining 36 patients. There was no statistically significant difference in the distribution of bacteria strains between the three groups (Table 3). Three patients did not receive an adequate treatment (one in the C group and two in the C-L group), all due to the presence of methicillin resistant *Staphylococcus aureus*. In addition, in the C-L group, one *P. aeruginosa *was resistant to quinolones.

**Figure 1:**
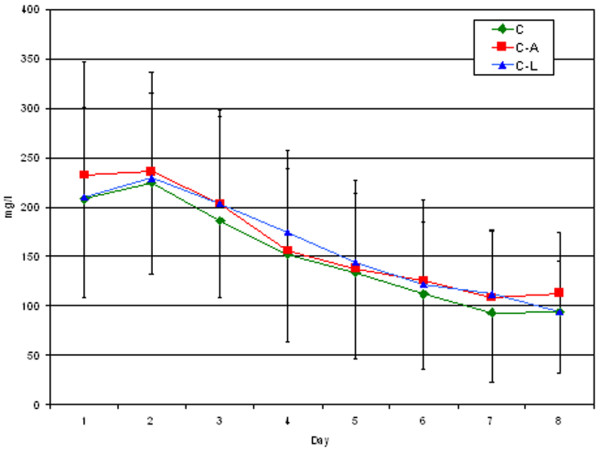
CRP time course. C, cefepime group; C-A, cefepime with amikacin group; C-L, cefepime with levofloxacin group

There were no significant differences in the evolution of PaO_2_/FiO_2_, temperature, leukocytosis and CRP level between the three groups. The corresponding values at day 1 and day 8 are given in Table 4 and the CRP evolution is shown in Figure 1. When the 15 excluded patients were added to the analysis, there was still no difference between groups and the evolution was the same.

### Outcome

Within 3 to 5 days after the start of therapy, new endotracheal samples were obtained from 70% of group C, 89% of group C-A and 85% of group C-L: the same bacteria as those found on day 1 were still present in the sputum of 8 patients in group C, 4 in group C-A and 12 in group C-L. After 7 to 10 days, persistence was documented in 4 patients out of 16 in group C, 5 out of 18 in group C-A, and 3 out of 13 in group C-L. New bacteria strains requiring new treatment were found in one patient in group C (one *P. aeruginosa*), three patients in group C-A (two *P. aeruginosa *and one *Enterobacter aerogenes *with extended spectrum beta-lactamase) and in three patients in group C-L (one *Proteus mirabilis*, one *Serratia marcescens *and one methicillin-resistant *S. aureus*).

**Figure 2:**
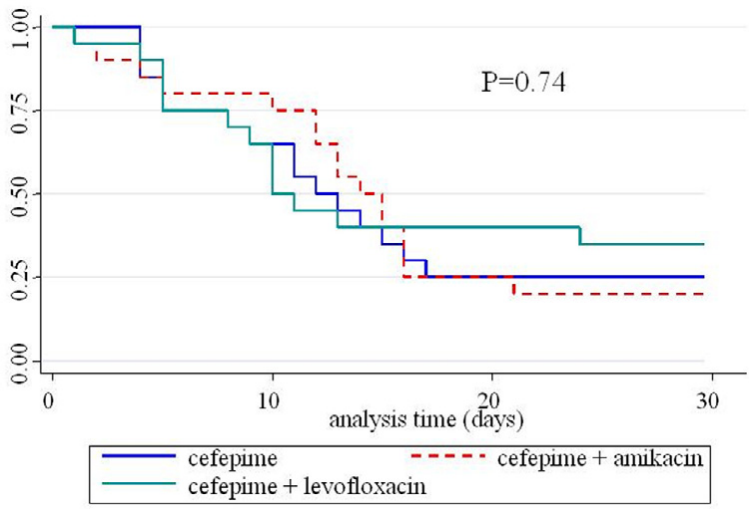
Kaplan-Meier curves of mechanical ventilation duration.

The length of ICU stay after the occurrence of infection was not different between the three groups: the medians (and 25th to 75th percentile in parentheses) were 15 (7.5 to 24.75), 16 (9 to 21) and 14 days (9.5 to 21.5) for groups C, C-A and C-L, respectively. There was also no difference between VFDs within 28 days after infection: the number of VFDs for each group was 16.1 ± 8.3 VFDs after C treatment, 12.6 ± 8.1 VFD after C-A treatment and 12.6 ± 10.4 VFD after C-L treatment (*p* > 0.05). Figure 2 shows the Kaplan-Meier curves of mechanical ventilation duration for the three groups (*p* > 0.05).

Ten patients died within 28 days, 2 in the C group (10%), 4 in the C-A group (21%) and 4 in the C-L group (20%). Only one death was clearly attributable to infection: one post-cardiac surgery patient who developed bronchopneumonia due to *P. aeruginosa *and died in septic shock with severe myocardial depression at day 3 post infection in group C. Dying patients had a more elevated CRP level at the eighth day compared to survivors in the three groups: 145 ± 53 mg/l versus 93 ± 54.9 mg/l (*p* = 0.033). Among the 15 patients with no microbiologically confirmed VAP, there was 1 other death within 28 days, in the C-A group.

## Discussion

The aim of this study was to compare monotherapy versus combination therapy for the treatment of VAP and to look for differences in the clinical and inflammatory parameters, including CRP level. This is the reason why we optimised the doses of antibiotics as recently recommended [25], and took a highly selected subgroup of ICU patients, represented mainly by post-trauma, postoperative or post-intracranial haemorrhage patients without infection at their entry into the ICU. This choice was made in order to obtain as far as possible a group of patients who did not have any life-threatening conditions other than VAP.

The diagnosis of VAP remains controversial. Some experts recommend obtaining quantitative culture of a protected specimen of pulmonary secretions [18,19] while others accept the use of the CPIS score based on clinical data [9,20,21]. However, this score is far from sensitive and also has a low specificity, as recently published [22,23]. In particular, the recent retrospective multicenter French study of Luyt and colleagues [24] comparing CPIS and quantitative culture in patients with VAP found a specificity as low as 47%. Due to the fact that quantitative cultures are not routinely available in our hospital, we used a modified CPIS to confirm the presence of VAP, in which we incorporated the rapid recent increase of CRP level, as it is one of our current diagnostic tools, and enrolled patients before obtaining the culture results. As such, all 59 patients with confirmed VAP had a CPIS above 6 with at least a positive non-quantitative culture. It is interesting to note that in this study, patients fulfilling the clinical criteria of VAP but with an absence of pathogens in their lung secretions had a CPIS significantly lower than the others and, in contrast, all patients with quantitative cultures had a CPIS of more than 6. It would be worth validating this 'new' CPIS in another prospective study.

The association of two drugs in the empirical treatment of VAP is recommended in order to increase the spectrum of activity, to decrease the emergence of resistance during treatment, and to improve the bactericidal activity of therapy [25]. If the first reason is obvious because of the presence of potential nosocomial resistant pathogens and cannot be questioned, the two others remain a matter of debate. Cefepime, a fourth generation cephalosporin, possesses bactericidal activity against the great majority of nosocomial pathogens in our hospital and covers almost 100% of non-inducible *Enterobacteriaceae*, more than 90% of inducible *Enterobacteriaceae *and nearly 90% of *P. aeruginosa*. We decided, therefore, to use it as a first line therapy for VAP, in association or not with either amikacin or levofloxacin, two other antibiotics with excellent activity against nosocomial pathogens. The combination of an anti-*Pseudomonas *beta-lactam with either an aminoglycoside or a quinolone such as levofloxacin or ciprofloxacin is indeed part of the published guidelines for the empirical treatment of VAP [25].

Among inflammatory parameters, CRP was chosen because of its routine use and its well known time course evolution. Interleukin 6 or procalcitonin, two other inflammatory parameters, well described as markers of severity, have not been validated as markers of response to antibiotic therapy [26,27]. CRP, however, which is used as a non-specific marker of inflammatory events and of bacterial infection, has been used to monitor response to antibiotic treatment [11]. Recently, the evolution of CRP levels has been described after CAP. Hansson and colleagues [16] showed that the mean time to a 50% decrease in CRP level was 3.3 days after CAP. Our results recorded in VAP patients, however, show that the decrease in CRP level is much slower (six days to obtain a 50% reduction). Of course, critically ill patients often have causes other than just a lung infection that increase their CRP levels. The vast majority of patients included in this study were either post-trauma or postoperative and their infections were a new insult but not the first they had encountered; however, the time delay of more than nine days between the hospital entry and the occurrence of infection (Table 2) should have been long enough to allow the CRP level, which usually peaks at 48 hours after trauma or surgery, to decrease. Figure 1 shows that the CRP levels peaked the second day after inclusion for VAP and confirms the new insult caused by the infection. The plasma half-life of CRP is about 19 hours and seems to be constant whatever the disease [28]; this means that the sole determinant of CRP level is the synthesis rate, which can be sustained after infections complicating trauma or surgery. The circulating CRP concentration decreases if the infectious stimulus ceases, however, and this should fall more rapidly with a better treatment than with a weaker one. This is what we were expecting to find in this study, but this was not the case: CRP levels failed to show any differences between monotherapy and combination therapy. The CRP levels decreased in the same manner in all three groups.

With regard to temperature, leukocytosis and PaO_2_/FiO_2_, Dennesen and colleagues [29] already described the resolution of infectious parameters after initiation of appropriate therapy for VAP in 27 patients. They showed that improvements in temperature, leucocytosis and PaO_2_/FiO_2 _were slow and most evident during the first six days [29].

There was no difference in terms of mortality, length of stay and VFDs between the three groups. Moreover, persistence of bacteria at the end of treatment was not reduced by combination therapy and recurrence of infection was as frequent in the three groups. The persistence of bacteria in tracheal aspirates confirms the observation of Dennesen and colleagues [29], who observed 50% persistence for *Enterobacteriaceae *and 100% persistence for *P. aeruginosa *in spite of appropriate therapy.

Weaknesses of this study include a lack of blinding of administration to therapy and the relatively small number of patients. We enrolled only 74 patients within 21 months, although we had to treat more than 300 ICU acquired pulmonary infections during this period. This was mainly due to the exclusion criteria, which did not admit patients receiving antibiotic treatment for another infection or infection already treated before randomisation. These criteria excluded all patients ventilated for CAP or for abdominal sepsis and all immunocompromised patients receiving antibiotics, among others. If this study failed to demonstrate any advantage of combination therapy over monotherapy, the use of empirical combination therapy remains probably relevant in patients with potentially multiresistant strains, as recommended by Chastre [30]. Importantly, the size of the samples in this study does not allow us to draw definitive conclusions regarding this issue.

## Conclusion

We failed to demonstrate any advantage of combination therapy over monotherapy. None of the recorded parameters, including time course of clinical and inflammatory parameters, duration of mechanical ventilation, ICU length of stay, bacteriological response and mortality, were influenced by the type of treatment. The mean time to obtain a 50% decrease in CRP levels after VAP was about six days, twice that seen after CAP, reflecting in part the complexity of the different insults ICU patients often may suffer from.

## Key messages

• 	The beneficial effects of an empirical combination antibiotic therapy for VAP were not confirmed in terms of the evolution of clinical and biological parameters.

• 	CRP plasma levels decreased by 50% within six days after antibiotic treatment of VAP.

## Abbreviations

CAP = community-acquired pneumonia; CPIS = clinical pulmonary infection score; CRP = C-reactive protein; ICU = intensive care unit; SOFA = sequential organ failure assessment; VAP = ventilator-associated pneumonia; VFD = ventilatory free days.

## Competing interests

The authors declare that they have no competing interests.

## Authors' contributions

PD and MM originated the study and contributed to the analysis of data. PD, JCP and CG prepared the paper. CG and MN collected the data, and JLC, DL and JCP enrolled the patients. All authors read and approved the final manuscript.

## Figures and Tables

**Table 1:** Modified clinical pulmonary infection score

	Number of points
Temperature (°C)	
≥ 36.5 and ≤ 38.4	0
≥ 38.5 and ≤ 38.9	1
≥ 39 and ≤ 36	2
Blood leucocytes (mm^3^)	
**≥ **4,000 and ≤ 11,000	0
<4000 or >11,000	1
Tracheal secretions	
Absence of tracheal secretions	0
Presence of non purulent tracheal secretions	1
Presence of purulent tracheal secretions	2
Oxygenation: PaO_2_/FiO_2 _(mmHg)	
**≥ **240 or ARDS	0
≤ 240 and no ARDS	2
Pulmonary radiography	
No infiltrate	0
Diffuse (or patchy) infiltrate	1
Localized infiltrate	2
CRP evolution (mg/l)	
Increase of >50 mg/l and <100 mg/l within the last 24 hours	1
Increase of >100 mg/l within the last 24 hours	2
Gram stain of tracheal secretion	
Bacteria visible	1

ARDS, acute respiratory distress syndrome; CRP C-reactive protein. PaO_2_/FiO_2_: arterial oxygen tension/inspiratory oxygen fraction

**Table 2:** Clinical and demographic data

Characteristic	Cefepime	Cefepime-amikacin	Cefepime-levofloxacin
Number of evaluable patients	20	19	20
Mean age (years)	53.1 ± 22.1	64.7 ± 19.1	59.2 ± 14.8
Gender (male/female)	13/7	10/9	15/5
Trauma	9	9	5
Cardiac surgery	4	5	6
Postoperative respiratory failure	2	1	4
Intracranial bleeding	3	3	4
Cardiac arrest	1	1	0
Haemorrhagic shock	1	0	1
ICU LOS (days): mean ± SD (median)	26 ± 23.1(21)	23 ± 7.9 (22.5)	34.7 ± 53.4 (20)
Hospital LOS before VAP (days): mean ± SD (median)	9 ± 6.4 (7)	11.3 ± 9.1 (8)	12.2 ± 12.3 (9)
Mean length of ventilatory support before VAP (days): mean ± SD (median)	7.2 ± 6.1 (6)	6.5 ± 2.1 (6)	9.4 ± 10.7 (5)
Mean APACHE II score	17.1 ± 4.6	14.6 ± 6.8	16.5 ± 6.4
SOFA score at day of infection	6.9	7	7.3
SOFA MAX	8.7 ± 3.3	9.6 ± 3.7	10 ± 3.3

ICU, intensive care unit; LOS, length of stay; SD, standard deviation; SOFA, sequential organ failure assessment; VAP, ventilator acquired pneumonia.

**Table 3:** Percentage of bacteria found from endotracheal aspirates in each treatment group

Bacteria	Cefepime -	Cefepime-amikacin -	Cefepime-levofloxacin -
*S. pneumoniae*	3.3	8.3	7.1
*Haemophilus influenzae*	6.7	4.2	7.1
*S. aureus*	23.3 (1)	25	28.6 (2)
*E. coli*	10	8.3	10.7
*Klebsiella *spp.	10 (1)	12.5 (1)	3.6 (1)
*Proteus *spp.	3.3	8.3	0
*Enterobacter *spp.	10	8.3	14.3 (1)
*Serratia marcescens*	6.7	12.5	7.1
*P.aeruginosa*	23.3 (1)	8.3 (1)	17.9
*Acinetobacter baumannii*	3.3	4.2	3.6

Numbers in parentheses correspond to the number of patients having positive blood culture with the same organism.

**Table 4:** Evolution of inflammatory parameters and oxygenation

Group	Day	PaO_2_/FiO_2 _(mmHg)	CRP (mg/l)	Temperature (°C)	Leukocytosis (10^3^/mm^3^)
C	1	173 ± 88	209 ± 100	38.6 ± 0.9	14.2 ± 5.8
	8	304 ± 114	94 ± 62	37.8 ± 0.7	15.5 ± 5.1
C-A	1	194 ± 95	233 ± 115	38.3 ± 0.9	14.3 ± 6.3
	8	253 ± 72	113 ± 61	37.7 ± 0.6	12.4 ± 6.4
C-L	1	176 ± 87	211 ± 90	38.8 ± 0.9	13.8 ± 6
	8	243 ± 72	94 ± 51	37.6 ± 0.7	15.1 ± 7.9

Groups: C, cefepime; C-A, cefepime with amikacin; C-L, cefepime with levofloxacin. CRP, C-reactive protein. PaO_2_/FiO_2_: arterial oxygen tension/inspiratory oxygen fraction
